# Clinical characteristics and CT features of hepatic epithelioid haemangioendothelioma and comparison with those of liver metastases

**DOI:** 10.1186/s13244-021-01143-x

**Published:** 2022-01-20

**Authors:** Xiaopeng Wang, Pan Liang, Peijie Lv, Rui Li, Ping Hou, Jianbo Gao

**Affiliations:** Department of Radiology, The First Affiliated Hospital of Zhengzhou University, No. 1, East Jianshe Road, Zhengzhou, 450052 Henan Province China

**Keywords:** X-ray computed tomography, Hepatic epithelioid haemangioendothelioma, Liver metastases

## Abstract

**Background:**

To analyse clinical characteristics and computer tomography (CT) findings of hepatic epithelioid haemangioendothelioma (HEH) and to determine differential features compared with liver metastasis (LM).

**Methods:**

This retrospective study included 80 patients with histopathologically confirmed HEH (*n* = 20) and LM (*n* = 60) of different primary tumours who underwent dynamic contrast-enhanced CT scans. CT findings included the location, contour, size, number, margin, and density of lesions, the patterns and degree of contrast enhancement of lesions, vascular invasion and changes in other organs. The enhancement ratio (ER) and tumour-to-normal parenchyma ratio (TNR) were calculated. Receiver operating characteristic curves (ROCs) were used to determine areas under the curve (AUCs).

**Results:**

About 65% of HEH lesions were located in submarginal areas. Significant differences were observed between HEH and LM patients in age, sex, and tumour marker positivity (*p* < 0.05). HEH showed minimal to slight enhancement, thin ring-like enhancement in arterial phase, and slight, homogeneous, progressive enhancement in the portal phase. HEH presented capsule retraction, and the “target” sign and the “lollipop” sign were significantly more frequent than in LM (*p* < 0.05). The ER and TNR in the arterial phase of HEH were lower than those of LM (*p* < 0.05). AUCs of ER and TNR in the arterial phase were 0.74 and 0.73, respectively.

**Conclusion:**

Lesions in subcapsular locations, capsular retraction, slight and thin ring-like enhancement, “target” and “lollipop” signs and lower ER and TNR in the arterial phase may represent important features of HEH compared with LM.

## Keypoints

- Dynamic CT imaging reveals typical features of HEH, including subcapsular location and nodule coalescence, capsular retraction, intralesional calcifications, and ring-like enhancement.
- When the typical “target” sign and “lollipop” sign are present, HEH should be highly suspected.
- When combined with clinical data, a lower AER and TNR, and CT imaging can help to improve differential diagnosis of HEH.

## Background

Epithelioid haemangioendothelioma usually arises in soft tissue and rarely occurs in the liver. Hepatic epithelioid haemangioendothelioma (HEH) is a rare primary vascular tumour with low to intermediate malignancy [1]. HEH, with a prevalence of 1 per 1,000,000 individuals [2], was first described by Weiss and Enzinger in 1982 [3]. In 1984, Ishak et al. [4] reported a series of HEHs and collected 32 cases from the literature. HEH is predominant in young and middle-aged women, though aetiologic factors remain unclear. Moreover, clinical and laboratory examinations of HEH are frequently non-specific. Given the variable clinical features and absence of useful diagnostic serum tumour markers, approximately 60% to 80% of HEHs are initially misdiagnosed [5]. Nevertheless, long-term survival in HEH is possible after successful liver resection or transplantation, even with the appearance of extrahepatic involvement [6]. Hence, it is important to diagnose HEH early and differentiate it from other malignant hepatic tumours before it multiple lesions develop. Currently, imaging plays an important role in diagnosis. There are many reports on the imaging characteristics of HEH, including those obtained via ultrasound (US), computer tomography (CT), magnetic resonance imaging (MRI) and positron emission tomography/computer tomography (PET/CT) [7–10]. Most HEHs show multiple lesions and minimal to slight and king-like enhancement, and these features are similar to those of liver metastases (LMs); therefore, HEH is often misdiagnosed as LM. Because LM is more invasive than HEH, with poor prognosis and different treatment strategies, understanding its imaging features will help to reduce difficulty in differential diagnoses in the clinic. To our knowledge, no studies have reported imaging signs for differentiating HEH from LM through in-depth quantitative data-based assessment. Because of its usefulness in a variety of situations and ready availability, CT has been recommended as part of the initial workup for many tumours and subsequent surveillance for metastatic disease after a diagnosis of primary cancer during the same examination [11]. Thus, the purpose of this study was to investigate clinical characteristics and CT features of HEH and assess their value in differentiating HEH from LM. In general, comparison of follow-up images has powerful value for selecting HEH treatment plans and evaluating prognosis.

## Methods

### Patients

**Fig. 1:**
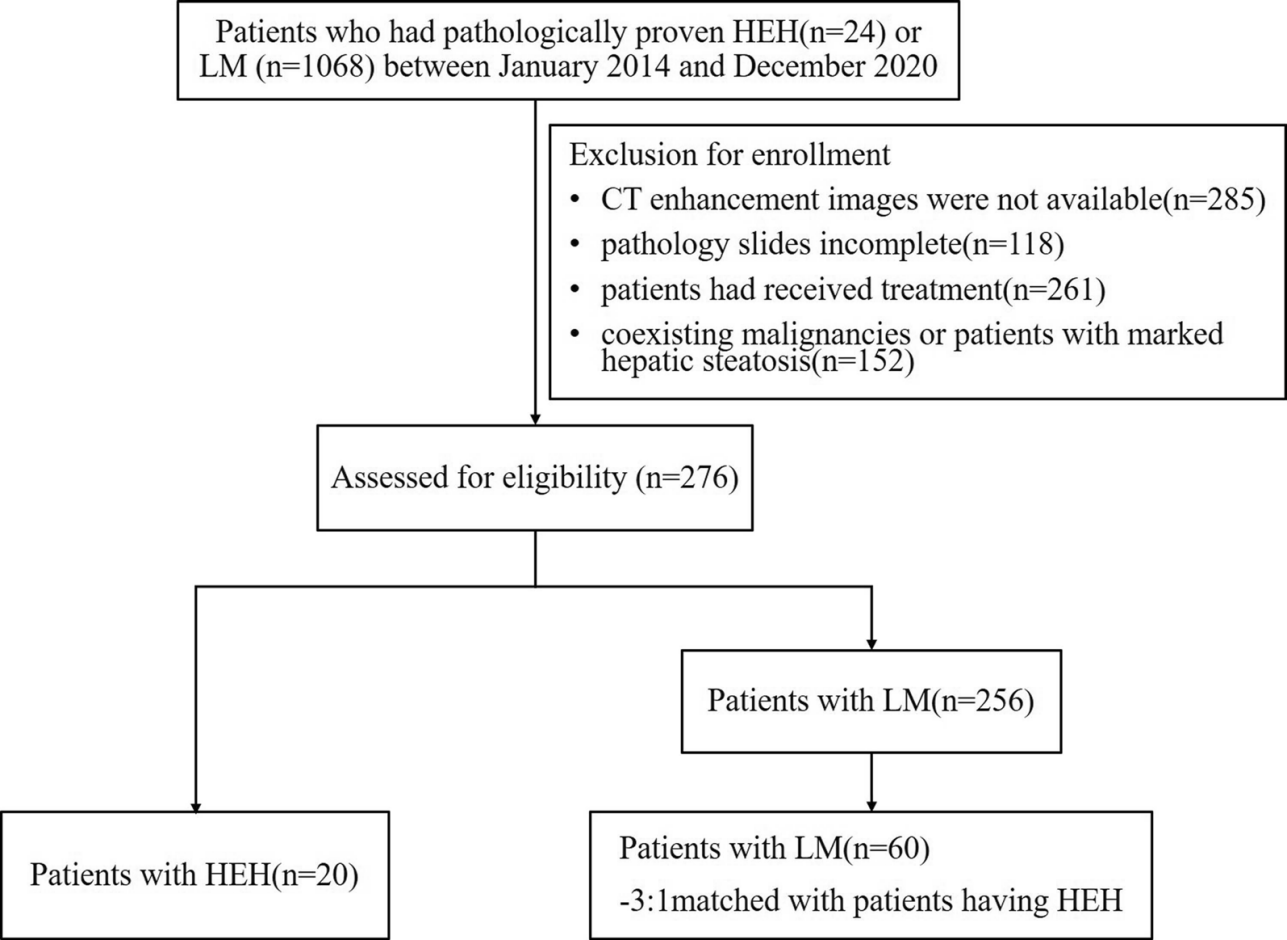
A summary flowchart of the study population. *HEH* hepatic epithelioid haemangioendotheliomas, *LM* liver metastasis

We retrospectively collected 24 patients with HEH and 1068 patients with LM from different primary tumours, as confirmed by pathology, between January 2014 and December 2020. All clinical and imaging data were obtained from the hospital information system (HIS) and picture archiving and communication system (PACS). The inclusion criteria were as follows: (1) patients who underwent biopsy or surgical resection for pathological results such as ''HEH''; (2) histopathological proof of at least one LM, and the primary disease of LM was also confirmed by pathology; and (3) patients who underwent a dynamic contrast-enhanced CT scan within 20 days prior to surgery or biopsy. The exclusion criteria were as follows: (1) pathology slides inadequate or unavailable for review; (2) patients who had incomplete or inadequate CT images, images of insufficient image quality, missing medical charts; (3) patients who had received treatment (including local therapy, systemic chemotherapy, or hepatectomy) prior to the CT scan; and (4) coexistence with other malignancies or patients with marked hepatic steatosis. A total of 276 patients (HEH = 20, LM = 256) met the criteria; however, there was a larger difference in number between the groups. Of the 256 patients with pathologically proven LM, 60 were selected, resulting in a matched control group at a ratio of 3:1 to the patients with HEH. The groups were matched in terms of year distribution, age range and classification of intrahepatic lesions. Ultimately, 80 patients (HEH = 20, LM = 60) comprised our study population (Fig. 1). The underlying primary extrahepatic malignancy of LM included colorectal cancer (*n* = 21), gastric cancer (*n* = 13), pancreatic cancer (*n* = 10), breast cancer (*n* = 5), lung cancer (*n* = 4), adenocarcinoma of the duodenum (*n* = 2), extrahepatic bile duct cancer (*n* = 2), ampulla of Vater cancer (*n* = 1), gallbladder cancer (*n* = 1), and renal cell carcinoma (*n* = 1).

### Scan protocol

Non-enhanced and dual-phase dynamic contrast-enhanced CT scans of the abdomen were performed for all patients using a 16-channel multi-detector CT scanner (Brilliance 16, Philips Medical Systems, Cleveland, OH, USA) or a 64-channel multi-detector CT scanner (Discovery CT750 HD CT Scanner, GE Healthcare Milwaukee, WI, USA). The parameters of the Brilliance 16 scanner were as follows: detector collimation, 1.5 mm; pitch, 1.25:1; tube voltage, 120 kVp; tube current, 80–270 mAs; rotation time, 0.6 s. The parameters of the Discovery CT750 scanner were as follows: detector collimation, 0.625 mm; pitch, 1.375:1; tube voltage, 120 kVp; tube current, 80–270 mAs; rotation time, 0.5 s. Dynamic contrast enhancement was performed by intravenous administration of 1.5 mL/kg iodinated contrast agent (350 mg I/mL) at a rate of 3.5 mL/s using an automatic bolus-tracking technique. After the injection, arterial phase scans were started 10 s after the attenuation threshold of the descending thoracic aorta reached 100 Hounsfield units. Portal phase scanning was performed 30 s after the arterial phase.

### Clinical analysis, treatment and follow-up

A clinical attending physician with 5 years of experience retrospectively reviewed the clinical data, including age, sex, chief complaint, and laboratory examination. Chief complaints were classified as abdominal discomfort or pain, fever, weak, weight loss, poor appetite, melena, haematemesis, asymptomatic and other symptoms. Laboratory examinations were as follows: liver function, tumour markers, and tumour abnormal protein (TAP). Clinical treatment and follow-up results were recorded for the patients.

### Image analysis

All CT images were analysed by two imaging diagnosticians (with 10 and 12 years of abdominal imaging experience) independently using a PACS. Although the readers were aware of the alternative diagnoses of HEH or LM, they were blinded to whether each lesion belonged to the HEH or LM group and to the clinical information and histopathological results. After independent image analysis, interobserver agreement was assessed for the evaluated CT imaging parameters; disagreements were resolved by consensus after reassessment of the images by the readers together. All images were analysed with regard to the following: (1) basic aspects—the maximum diameter (on axial images), number and classification (solitary nodular type, multifocal nodular type, and diffuse type), contour (round, round-like or strike-like, regular or irregular), margin (ill- or well-defined), location (scattered distribution, liver subcapsular) and density of the lesion (homogeneous or heterogeneous, hypo-, iso- or hyperdense, relative to the adjacent normal liver parenchyma) and the presence of a coalescence of lesions in cases of multiple lesions; (2) special signs and enhancement features [10]—(1) subcapsular location (any portion of the lesion in contact with the liver capsule); (2) capsular retraction (indentation of the hepatic contour for lesions with a subcapsular location); (3) rim-like (thin or thick peripheral enhancement encircling the lesion); (4) peripheral nodular enhancement (enhanced peripheral nodular areas); (5) “target” sign [12] (minimally enhanced centre and an enhanced inner peripheral rim juxtaposed with a minimally enhanced outer rim); (6) “lollipop” sign [12] (compression displacement or truncation of adjacent vessels around the lesion in portal venous phase); (7) calcification (lesions showing point, nodular or irregular high density, similar to that of bone); and (8) abnormal perfusion in the arterial phase (hepatic parenchyma around lesions showing patchy enhancement in the arterial phase and disappearance in the portal venous phase). When evaluating the outline and size of intrahepatic lesions, the observer should fix the abdominal window (window width 240 HU, window level 40 HU), whereas the location, shape, enhancement feature and other signs of the lesions can be appropriately adjusted according to the situation.

Quantitative analysis was performed by measuring the attenuation of the lesion, and the normal parenchyma (Hounsfield units, HU) in the arterial and portal phases was measured in a circular or irregular region of interest (ROI). First, a slice showing the maximum diameter of the lesion on the axial image was selected. The ROI was then manually drawn within the boundary of the tumour, attempting to contain the whole tumour as much as possible. For all measurements, the size and shape of the ROI were kept consistent between the two phases, and the information from three lesion images of multiple lesions (where the lesion diameter was between 2 and 5 cm) was obtained to calculate the average value. The means were then used to calculate the arterial enhancement ratio (AER) of the tumour (HU arterial—HU plain/HU plain), portal enhancement ratio (PER) (HU portal—HU plain/HU plain) [13], arterial tumour to normal parenchyma ratio (ATNR) (HU arterial/HU liver), and portal tumour to normal parenchyma ratio (PTNR) (HU portal/HU liver) [14]. The enhancement degree during contrast-enhanced imaging can be divided into minimal enhancement (CT attenuation changes less than 10 HU), slight enhancement (CT attenuation changes between 10 and 20 HU), or progressive moderate enhancement (CT attenuation changes more than 20 HU) [15].

### Histopathologic examination

All HEH cases were determined by histopathology, including needle biopsy under ultrasound (*n* = 2) or CT (*n* = 14) guidance and surgical resection (*n* = 4). Each case of HEH was diagnosed based on light microscopic examinations, including haematoxylin–eosin and immunohistochemical staining results, confirming the endothelial origin of the tumour cells according to the tumour classification of the World Health Organization [16]. Microscopically, the tumours consisted of proliferated fibrous tissue intermingled with abundant vacuolated cytoplasm epithelioid cells. Endothelial markers (mainly factor VIII-related antigen, CD34, CD31, Ki-67 5%) were positive according to immunohistochemical staining. All LMs were confirmed by pathology, clinical medical history and CT/MRI imaging during follow-up.

### Statistical analysis

All statistical calculations were performed using Statistical Package for the Social Sciences (SPSS 20.0, Chicago, IL, USA). Categorical variables, including clinical characteristics (e.g., sex, main symptoms, laboratory examination) and qualitative CT features (e.g., classification, location, contour, border, density, enhancement pattern and degree, special signs, extrahepatic involvement), are described as frequencies or percentages. The χ^2^ test (Pearson and continuity correction) and Fisher’s exact test were employed to evaluate differences between the groups. Continuous variables (age, ER and TNR) that followed a normal distribution are reported as the means and standard deviation and were compared using Student’s *t*-test (including the corrected *t*-test). Receiver operating characteristic (ROC) curves of ER and TNR were obtained to generate the area under the curve (AUC) and an optimal cut-off, at which the sum of the sensitivity and the specificity was maximum. In general, an AUC between 0.5 and 0.7 suggests low diagnostic value, between 0.7 and 0.9 suggests medium diagnostic value, and between 0.9 and 1 suggests high diagnostic value. Sensitivity, specificity, and odds ratios (ORs) with 95% confidence intervals (CIs) were analysed for variables differing significantly between the two groups. Kappa statistics were applied to determine the interobserver agreement of each variable. The Spearman correlation test was used to analyse correlation among CT features, treatment and clinical results. A κ value of up to 0.20 was interpreted as slight agreement, 0.20–040 as fair agreement, 0.40–0.60 as moderate agreement, 0.60–0.80 as substantial agreement, and > 0.80 as almost perfect agreement. Statistical significance was defined by a *p* value < 0.05.

## Results

### Clinical information, treatment and follow-up

Table 1 summarises the clinical characteristics, treatments and follow-up results of the HEH patients (5 males and 15 females; median age, 41 and 42 years; range, 21–71). The basis for decision-making regarding treatment for HEH was the histopathologic classification, and the mode of hepatic involvement and the presence or absence of extrahepatic involvement were the main considerations. In general, the rate of progression, severity of signs and symptoms, and response to treatment methods should be considered for each patient. Treatment details were not available for 2 patients. The remaining 18 patients were managed with no treatment (3/20, 15%), liver resection (4/20, 20%), transcatheter arterial chemoembolisation (TACE) (2/20, 10%) and radiofrequency ablation (RFA) (1/20, 5%), chemotherapy (2/20, 10%), and complex therapy (chemotherapy, radiotherapy and immunotherapy) (6/20, 30%). During the follow-up, serial clinical and imaging information were available for 16 patients, which allowed evaluation of tumour progression. Of the remaining four patients, only telephone follow-up data were available for one, with no relevant imaging information, and the remaining 3 patients were lost to follow-up. At the last follow-up (4–62 months), 15 patients were alive; 1 patient had died due to underlying disease (digestive and respiratory diseases). Reduction, no obvious change, enlargement and new lesions were found in two, five, three and five patients, respectively, on the final CT images compared to the original CT images.

**Table 1:** Patients basic characteristics and clinical course of HEH

Case	Classification	Sex	Age(y)	Symptom	Liver function		Tumour mark			Treatment	Follow-up	
					ALT	AST	CA199	CA125	HBV		Time (m)	Outcome
1	Solitary	M	48	Abdominal pain	Normal	Normal	Negative	Negative	Negative	TACE	41	no change
2	Solitary	F	35	Asymptomatic	normal	Normal	Negative	Negative	Negative	Hemihepatectomy	4	no change
3	Solitary	F	41	Fever	Normal	Normal	Negative	Negative	Negative	Hemihepatectomy	10	new lesion
4	Multiple	M	42	Asymptomatic	93.58	73.26	negative	negative	Negative	no Treatment	25	alive
5	Multiple	F	47	Asymptomatic	normal	Normal	Negative	Negative	Negative	Hemihepatectomy	13	new lesions
6	Multiple	F	46	Asymptomatic	normal	Normal	Negative	Negative	positive	Hemihepatectomy	62	new lesions
7	Multiple	F	69	Weight loss	normal	Normal	Negative	124.18	negative	TACE	24	reduction
8	Multiple	F	31	Asymptomatic	normal	Normal	Negative	negative	negative	Complex therapy	36	no change
9	multiple	F	55	Weak	normal	Normal	Negative	negative	Negative	No treatment	26	new lesions
10	multiple	F	29	Asymptomatic	normal	Normal	Negative	negative	Negative	complex Therapy	22	new lesions
11	multiple	F	48	Abdominal pain	60.36	normal	negative	negative	negative	unknown	no	unknown
12	multiple	M	38	asymptomatic	88.31	normal	negative	negative	negative	chemotherapy	no	Lost to follow-up
13	diffuse	F	34	poor appetite	106.39	114.68	negative	negative	negative	chemotherapy	19	Enlargement
14	diffuse	M	36	asymptomatic	Normal	Normal	38.95	Negative	Negative	Complex therapy	18	Enlargement
15	diffuse	F	39	asymptomatic	98.17	73.28	negative	negative	Negative	Complex therapy	4	No change
16	diffuse	F	40	abdominal pain	Normal	normal	negative	48.26	Negative	No treatment	18	No change
17	diffuse	F	52	abdominal pain	Normal	normal	negative	62.52	Negative	RFA	10	Reduction
18	diffuse	F	37	abdominal pain	Normal	normal	negative	negative	Negative	Complex therapy	13	Enlargement
19	diffuse	M	71	abdominal pain	102.35	116.87	negative	162.36	Negative	Complex therapy	17	dead
20	diffuse	F	21	asymptomatic	Normal	Normal	Negative	Negative	Negative	Unknown	no	unknown

* HEH* hepatic epithelioid haemangioendothelioma; *M* Male; *F* Female; *ALT* glutamic-pyruvic transaminase, normal range:0–40. *AST* glutamic oxalacetic transaminase, normal range 0–40; *CA199* carbohydrate antigen 199, normal range 0.01‑37 U/ml; *CA125* antigen 125, normal range 0.01‑35U/ml; *HBV* hepatitis B virus; *TACE* transcatheter arterial chemoembolisation; *RAF* radiofrequency; *y* year; *m* month

### Imaging findings of HEH

All CT imaging findings and enhancement patterns of the HEH patients are summarised in Table 2.

**Fig. 2:**
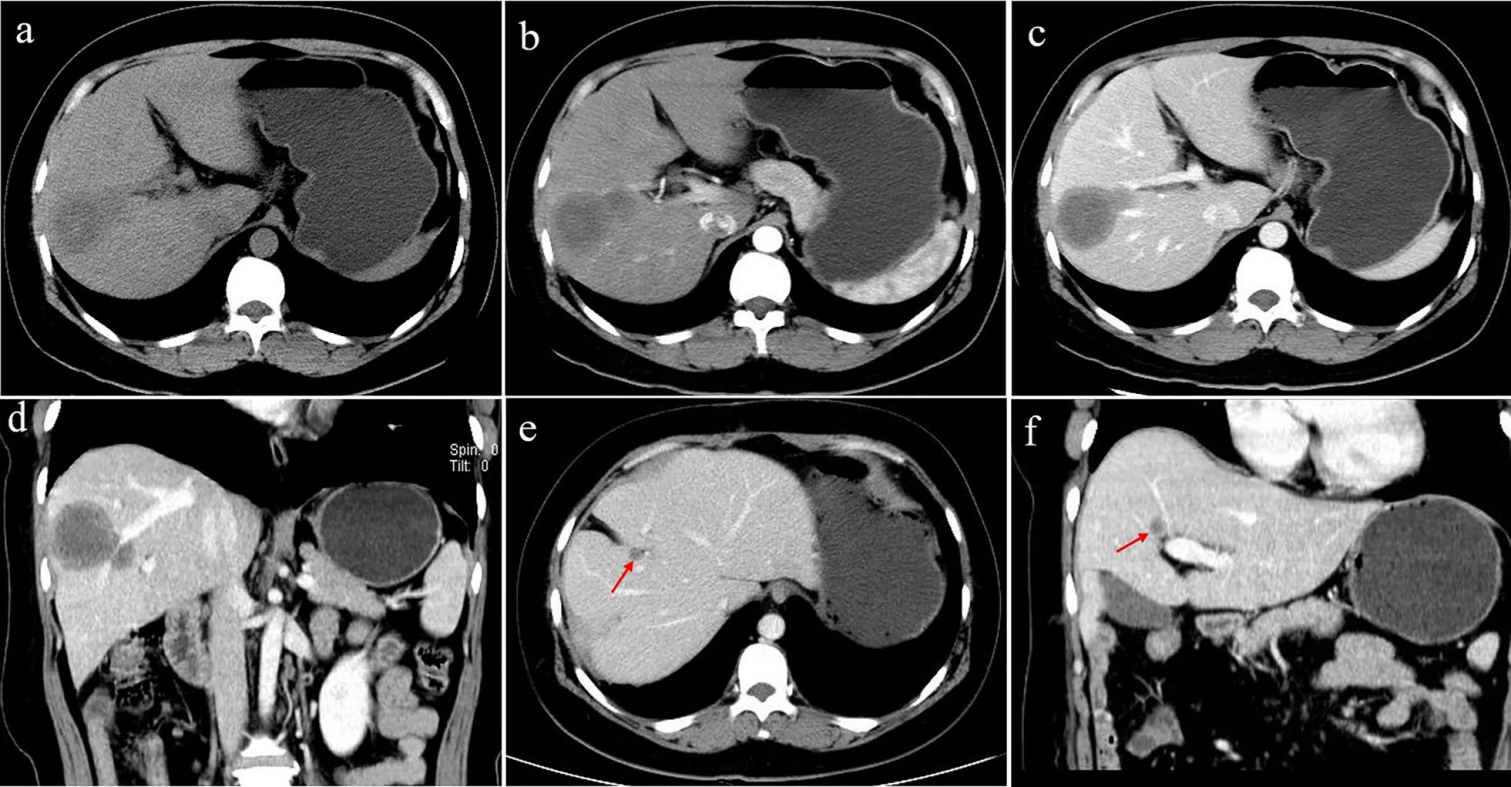
A 41-year-old female with solitary nodular type HEH. An axial unenhanced CT image **a** shows a round nodule with ill-defined, heterogeneous hypodensity. An axial arterial phase image (**b**) shows rink-like enhancement. An axial portal phase image (**c**) shows “target” sign enhancement. Coronal reconstruction of the portal venous phase (**d**) shows a narrowing portal vein branch adjacent to the lesion. This patient was initially misdiagnosed with hepatic abscesses. On follow-up CT performed 10 months after resection (**e**, **f**), a new lesion was detected in the right lobe of the liver (red arrowheads)

**Fig. 3:**
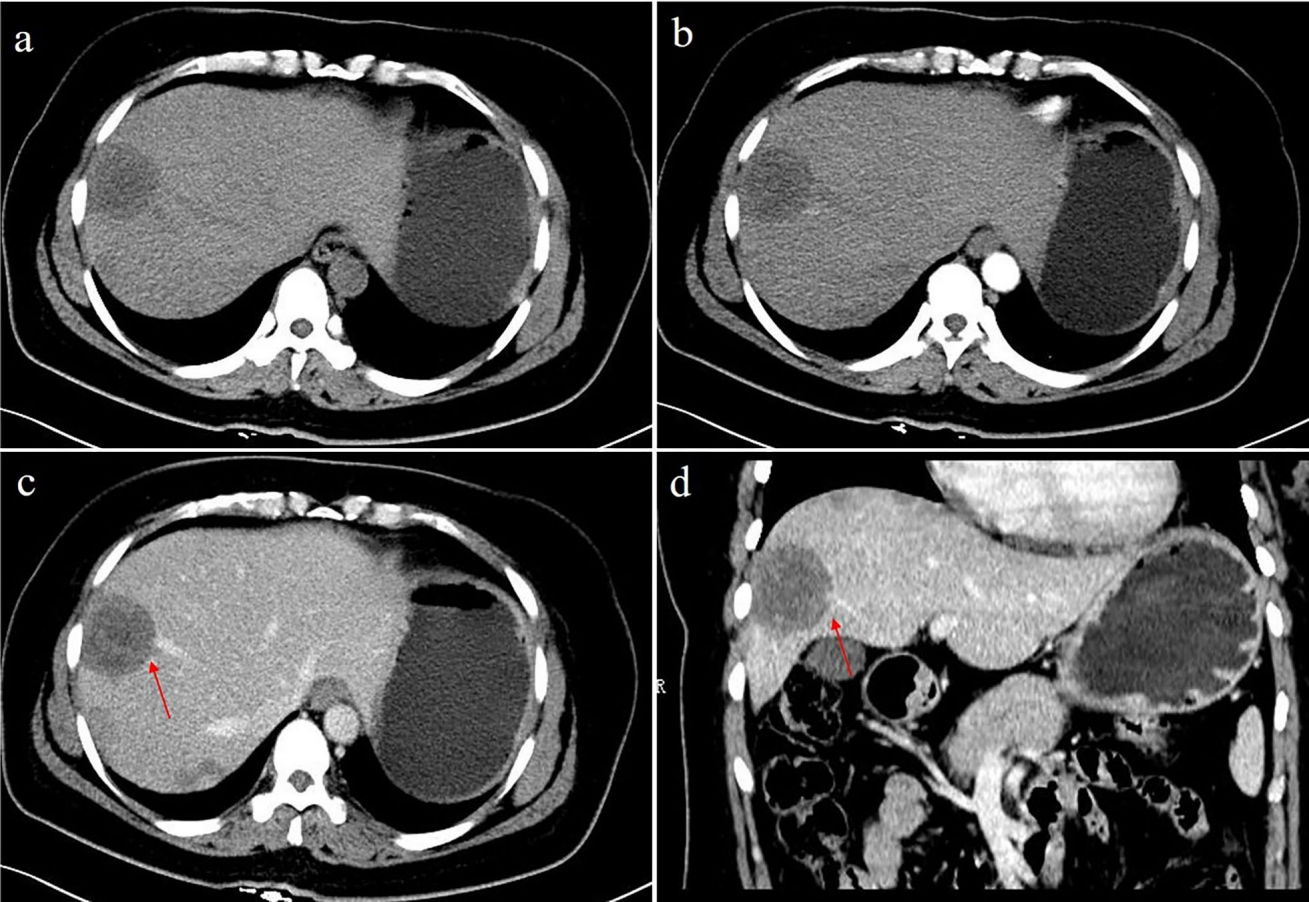
A 48-year-old female with multifocal nodular type HEH. An axial unenhanced CT image (**a**) shows multiple round-like low-density lesions in the right lobe of the liver. The lesions show slight enhancement in the arterial phase (**b**). Axial and coronal reconstruction of portal phase CT images (**c**, **d**) show a larger nodule in the right lobe with portal veins entering and terminating in the periphery of the lesion (red arrow). This configuration resembles a typical “lollipop” sign

**Fig. 4:**
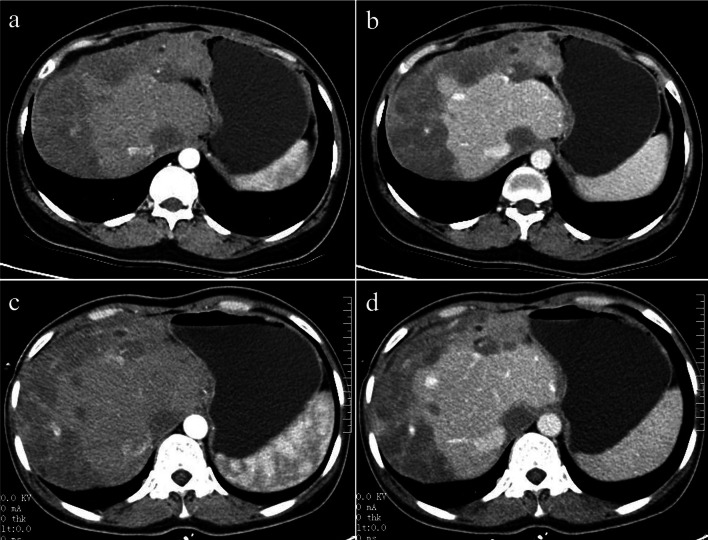
A 40-year-old female with diffuse nodular type HEH. Axial arterial and portal phase CT images (**a**, **b**) show multiple, diffuse nodules that lack obvious enhancement and confluent masses with strip-like enhancement located at the subcapsular region of the liver, accompanied by liver morphological changes and proportion imbalance. On follow-up CT performed 18 months later without any treatment, axial arterial and portal phase images (**c**, **d**) show no obvious changes in the lesions

**Fig. 5:**
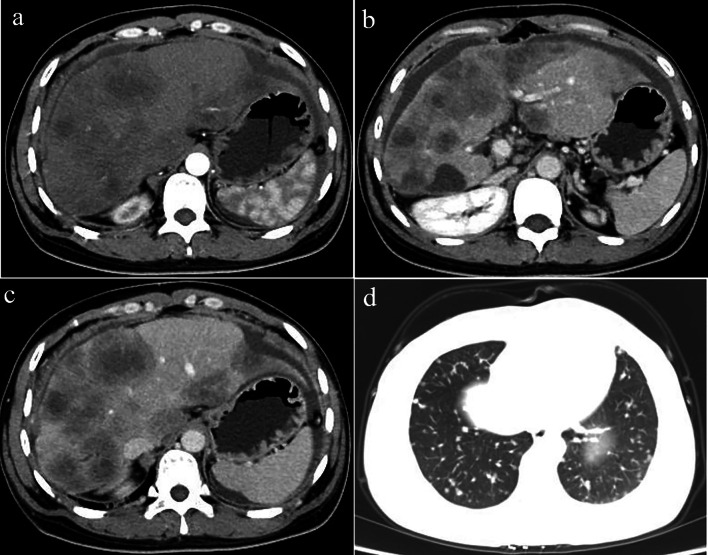
A 34-year-old female with diffuse nodular type HEH. Axial and multiple phase contrast-enhanced CT images (**a**–**c**) show multiple, different-sized, coalescing, homogeneous, hypoattenuating nodules diffusing throughout the liver parenchyma. Masses show ring-like enhancement and the “target” sign. At the same time, there was arc-shaped effusion around the liver and peritoneal thickening. Peripheral distribution of masses and capsular retraction are also observed. The axial lung window image (**d**) shows lung involvement consisting of multiple round-like nodules

**Fig. 6:**
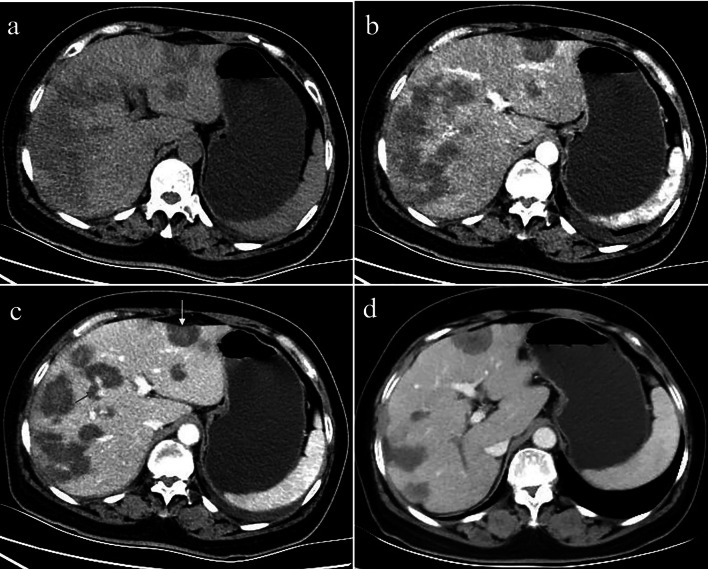
A 52-year-old female with diffuse nodular type HEH. Axial unenhanced CT images (**a**) show diffuse coalescing hypoattenuating lesions. Arterial phase CT images (**b**) show slight enhancement, and some nodules confluent to strip‑like or snowmelt are located subcapsularly in the liver. In the portal phase (**c**), the lesions are not progressively enhanced, a small branch of the portal vein is seen passing through the middle of the lesions (red arrow), and capsular retraction (yellow arrow). On follow-up CT performed 10 months after radiofrequency ablation, axial portal vein phase CT image (**d**) shows lesion reduction

1. The solitary nodular type was detected in 3 patients (3/20, 15%), including 3 solitary lesions, all of which were located in the right lobe of the liver. Only 1 patient showed heterogeneous density with spot calcification; the other patients showed homogeneous density. One patient exhibited peripheral nodular enhancement, with enlarged feeding arteries and hyper-perfusion in the arterial phase. A “target” sign and portal vein branch invasion were detected in one patient (Fig. 2).
2. The multifocal nodular type was detected in 9 patients (9/20, 45%). The lesions were located in the right lobe of the liver in only one patient; the whole liver was involved in all of the remaining 8 patients. Coalescent lesions were detected in 6 patients (6/9, 66.7%). One patient showed multiple nodules with calcifications; the other 8 patients showed hypoattenuation compared with the normal liver parenchyma on non-contrast CT imaging. More than 80% of the lesions were peripheral and extended to the liver margin. Retraction of the liver capsule overlying the tumour nodules was detected in 10 lesions of 6 patients. Six patients showed minimal and slight enhancement in the arterial phase; 5 patients showed thin ring-like enhancement. Four patients exhibited slight to moderate progressive centripetal enhancement in the portal phase. In 5 patients, “target” sign enhancement in the portal phase was detected. The “lollipop” sign was observed in 8 lesions of 4 patients (Fig. 3).
3. The diffuse type was observed in 8 patients (8/20, 40%). CT features included diffuse low-density lesions with minimal residual areas of normal liver parenchyma, multiple nodules and confluent masses with strip‑like enhancement located at the edge of the liver. Coalescent lesions were detected in most of the cases (7/8, 87.5%). Changes similar to those in liver cirrhosis were detected in 2 patients, such as liver morphological changes, proportion imbalance, and widened hepatic hilar and hepatic fissures (Fig. 4). Multiple lesions displayed slight or ring-like enhancement in the arterial phase and gradual homogeneous or heterogeneous enhancement in the portal phase. One patient showed involvement of lymph nodes; both the peritoneum and lung were invaded in another patient (Fig. 5). Stenosis or occlusion of the branches of the portal and hepatic veins was also detected in 2 patients (Fig. 6).

**Table 2:** CT imaging features and enhancement patterns of HEH

Case	Type	Diameter	Contour	Calcification	Patterns of contrast enhancement		Capsular	Lollipop	Vascular	Transference
					Artery phase	Portal venous phase	retraction	sign	invasion	
1	Solitary	47	Round	Spot	Nodular/hyper-perfusion	Heterogeneous progressive	Yes	No	No	Peritoneum
2	Solitary	29	Round	No	Slightly	Homogeneous slightly	No	Yes	No	No
3	Solitary	32	Round	No	Slightly	Target sign/moderate	No	No	Yes	No
4	Multiple	9–21	Round	No	Minimal	Homogeneous slightly	Yes	No	No	No
5	Multiple	23–41	Round	Nodular	Slightly	Target sign/slightly	Yes	no	no	no
6	Multiple	11–26	Round	No	Ring-like	Target sign/moderate	Yes	Yes	No	No
7	Multiple	5–69	Round	No	Ring-like/hyper-perfusion	Homogeneous moderate	No	No	No	No
8	Multiple	6–33	Round	No	Ring-like	Target sign/moderate	No	Yes	No	No
9	Multiple	9–26	Round-like	No	Slightly	Homogeneous/moderate	No	No	No	No
10	Multiple	3–62	Round	No	Ring-like	Target sign/slightly	Yes	Yes	No	lung
11	Multiple	6–53	Round	No	Slightly	Homogeneous slightly	Yes	Yes	No	No
12	Multiple	12–43	Round-like	No	Ring-like	Target sign/moderate	Yes	No	No	Lung, peritoneum
13	Diffuse	5–73	Round	No	Ring-like	Target sign/moderate	Yes	Yes	Yes	Lung, lymph gland
14	Diffuse	3–38	Irregular	No	Ring-like	Homogeneous moderate	Yes	Yes	No	No
15	Diffuse	8–89	Irregular	No	Ring-like/hyper-perfusion	Heterogeneous slightly	No	No	No	No
16	Diffuse	> 32	Strip‑like,	no	Minimal	Target sign/heterogeneous	No	Yes	Yes	No
17	Diffuse	23–69	Strip‑like,	no	Minimal	Homogeneous slightly	Yes	Yes	No	No
18	Diffuse	4–36	Irregular	No	Ring-like/hyper-perfusion	Target sign/slightly	Yes	Yes	No	No
19	Diffuse	5–73	Strip-like	No	Minimal	Homogeneous slightly	Yes	No	No	Peritoneum
20	Diffuse	16–57	Flaky	No	Minimal	Homogeneous slightly	Yes	No	No	No

*HEH* hepatic epithelioid haemangioendothelioma

### Correlation between CT features, therapeutic decisions and clinical outcomes of HEH

The classification, location, distribution, contour, border, density, enhancement pattern and special signs of the lesions did not correlate with therapeutic decisions or clinical outcomes. Lesion size was modestly associated with the therapeutic decision (correlation coefficient *k* = 0.566, *p* = 0.009). In addition, there was a certain correlation between the "target sign" and clinical outcome (correlation coefficient *k* = 0.504, *p* = 0.024).

### Comprehensive comparative analysis of HEH and LM

Table 3 summarises the comparison of clinical characteristics between HEH and LM. There were significant differences between the two groups in age, sex, main symptoms, and laboratory examination (*p* < 0.05). The majority of HEH patients were middle-aged women, most of whom had no obvious symptoms (50%), normal liver function (70%) and normal tumour markers (70%); the majority of LM patients were middle-aged and elderly, with no sex predilection, who had abnormal liver function (57%) and increased tumour markers (47%).

**Table 3:** Comparison of clinical characteristics between HEH and LM

Clinical characteristics	HEH (*n* = 20)	LM (*n* = 60)	*P* value
Age (year)^a^	42.95 ± 12.29	57.40 ± 9.50	0.000
Sex*			0.007
Male	5 (25)	36 (60)	
Female	15 (75)	24 (40)	
Main symptoms			
Abdominal discomfort/pain*	6 (30)	37 (62)	0.014
Fever*	1 (5)	3 (5)	1.000
Weak*	1 (5)	12 (20)	0.221
Weight loss*	1 (5)	42 (70)	0.000
Poor appetite*	1 (5)	25 (42)	0.002
Melena/hematemesis*	0 (0)	27 (45)	0.000
Asymptomatic*	10 (50)	8 (13)	0.002
Liver function abnormal*	6 (30)	34 (57)	0.039
Tumour mark positive*	6 (30)	28 (47)	0.035
CA 199	1 (5)	38 (63)	
CA125	4 (20)	39 (65)	
CEA	1 (5)	41 (68)	
Tumour abnormal protein rise*	1 (5)	49 (82)	0.000

*HEH* hepatic epithelioid haemangioendothelioma, *LM* liver metastases

^*^Data are number of patients with the percentage in parenthesis; *p* values were calculated from χ^2^ test and Fisher’ exact test

^a^Data are mean ± standard deviation; *p* values were calculated from student *t* test

Table 4 summarises the comparison of quantitative and qualitative CT findings between HEH and LM. A significant difference was observed in the distribution of tumour location (*p* < 0.000). Most of the HEH masses (13/20, 65%) were located in the subcapsular region of the liver, whereas LM had no obvious distribution characteristics. With regard to the lesion border, 13 (65%) of the 20 HEH masses were well defined, and 42 (70%) of the 60 LM were ill defined. Minimal to slight, thin ring-like enhancement in the arterial phase and slight enhancement in the portal phase were observed in most of the HEH masses; slight to moderate, thick ring-like enhancement in the arterial phase and moderate enhancement in the portal phase were observed in most LMs. In HEH, the frequencies of capsular retraction, the “target” sign, and the “lollipop” sign were significantly higher than those in LM (*p* < 0.05). The presence of extrahepatic involvement, including lymph node, peritonea and bone invasion, was detected in fewer patients with HEH than in those with LM (*p* < 0.05). All interobserver variability values were acceptable (κ coefficient range, 0.602–0.958).

**Table 4:** Comparison of quantitative and qualitative CT findings between HEH and LM

CT findings	HEH (*n* = 20)	LM (*n* = 60)	*P* value	*k* coefficient
Classification*			0.963	0.912
Solitary nodular type	3 (15)	8 (13)		
Multifocal nodular type	9 (45)	29 (49)		
Diffuse types	8 (40)	23 (38)		
Location*			0.000	0.897
Scattered distribution	7 (35)	51 (85)		
Liver subcapsular	13 (65)	9 (15)		
Contour*			0.138	0.846
Round/round-like	13 (65)	30 (50)		
Irregular	3 (15)	24 (40)		
Strip‑like/flaky	4 (20)	6 (10)		
Border*			0.005	0.851
Well defined	13 (65)	18 (30)		
Ill defined	7 (35)	42 (70)		
Density of plain scan*			0.639	0.803
Low	17 (85)	45 (75)		
Equal	1 (5)	6 (10)		
High	2 (10)	9 (15)		
Enhancement patter and degree in AP*			0.005	0.768
Minimal	5 (25)	3 (5)		
Slightly	5 (25)	13 (22)		
Moderate/obvious	0 (0)	17 (28)		
Ring-like	9 (45)	27 (45)		
Nodular	1 (5)	0 (0)		
Enhancement patter and degree in PVP*			0.068	0.755
Slightly	12 (60)	22 (37)		
Moderate	8 (40)	38 (63)		
Special signs				
Capsular retraction*	13 (65)	6 (10)	0.000	0.892
Target sign*	9 (45)	7 (12)	0.004	0.794
Lollipop sign*	10 (50)	3 (5)	0.000	0.813
Vascular invasion*	3 (15)	12 (20)	0.869	0.773
Calcification*	2 (10)	4 (7)	0.493	0.958
Extrahepatic involvement				
Lung*	3 (15)	16 (27)	0.448	0.742
Peritoneum*	3 (15)	26 (43)	0.022	0.679
Lymph node*	1 (5)	37 (62)	0.000	0.703
Bone*	0 (0)	7 (12)	0.026	0.602
AER^a^	0.26 ± 0.10	0.37 ± 0.13	0.001	
PER^a^	0.58 ± 0.13	0.61 ± 0.15	0.369	
ATNR^a^	0.69 ± 0.05	0.71 ± 0.04	0.022	
PTNR^a^	0.51 ± 0.04	0.53 ± 0.05	0.056	

*HEH* hepatic epithelioid haemangioendothelioma, *LM* liver metastases, *AER* arterial enhancement ratio, *PER* portal enhancement ratio, *ATNR* arterial tumour to normal parenchyma ratio, *PTNR* portal tumour to normal parenchyma ratio

^*^Data are number of patients with the percentage in parenthesis; *p* values were calculated from χ^2^ test and Fisher’ exact test

^a^Data are mean ± standard deviation; *p* values were calculated from student *t* test

**Fig. 7:**
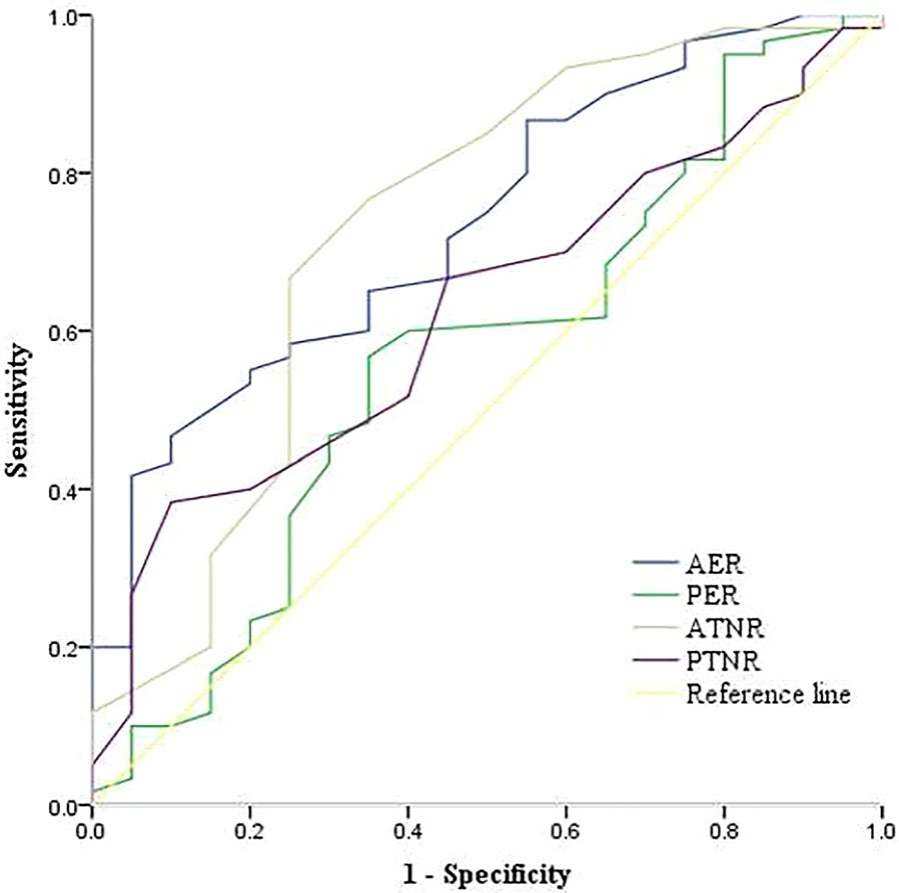
ROC curves for the arterial enhancement ratio (AER), portal enhancement ratio (PER), arterial tumour to normal parenchyma ratio (ATNR), and portal tumour to normal parenchyma ratio (PTNR). There were significant differences between the arterial enhancement ratio and the arterial tumour to the normal parenchyma

HEH masses had a significantly lower AER and ATNR than LMs (*p* < 0.05). Conversely, no significant differences in PER and PTNR were found. Using ROC analysis, cut-off values for AER, PER, ATNR and PTNR were 0.36, 0.63, 0.69 and 0.51, respectively; the corresponding AUCs (95% CI) were 0.74 (0.62–0.86), 0.57 (0.42–0.72), 0.73 (0.59–0.87), and 0.63 (0.49–0.76), respectively (Fig. 7). The above continuous variables were transformed into categorical variables according to the cut-off values. Table 5 summarises the sensitivity, specificity, and OR of each significant variable.

**Table 5:** Sensitivity and specificity of CT finding in diagnosis of HEH

CT findings	Sensitivity (%)	Specificity	OR (95%CI)
Liver subcapsular	65.0	85.0	10.52 (3.30–33.58)
Capsular retraction	65.0	90.1	16.71 (4.80–58.18)
Target sign	45.0	88.3	6.2 (1.90–20.20)
Lollipop sign	50.0	96.7	14.0 (3.67–53.50)
AER < 0.36	80.0	48.3	3.74 (1.12–12.51)
ATNR < 0.69	75.0	78.3	78.3 (3.32–35.44)

*HEH* hepatic epithelioid haemangioendothelioma, *AER* arterial enhancement ratio, *ATNR* arterial tumour to normal parenchyma ratio

## Discussion

HEH appears to have a clinical course between that of benign haemangioma and angiosarcoma. The World Health Organization classifies HEH as a malignant tumour of vascular origin [1]. Approximately 500 cases have been reported to date. HEH predominantly occurs in young and middle-aged women; the average age of the patients is 42 years old, and the female-to-male ratio is 1.6–2.0:1. The ratios of age and sex in this study are basically consistent with those reported in the literature [17]. The aetiology of HEH remains unknown; however, it may be associated with oral contraceptive use, exposure to polyethylene, alcohol, trauma or viral hepatitis [18]. Clinical manifestations of HEH are non-specific but most likely include upper abdominal discomfort, abdominal pain, poor acceptance, weight loss, jaundice, fever, and nausea [19]. Some diffuse and bilobar forms may show complicated clinical presentations, such as Budd-Chiari syndrome, portal hypertension or liver failure [5]. Biochemical examinations are frequently normal. In our study, 6 patients (6/20, 30%) exhibited a slight increase in aminotransferase and tumour markers. In contrast, most patients with LMs have obvious clinical symptoms (primary tumour- and cachexia-related), abnormal liver function, increased tumour markers and TAP. There was overall a significant difference between the two groups. Thus, a diagnosis of HEH may be considered for young and middle-aged women and asymptomatic patients with negative laboratory tests. LM mainly affected middle-aged and elderly individuals, with no significant sex predilection, and laboratory examinations were mostly abnormal.

CT imaging in our study also showed that the percentage of solitary nodular type cases was lower than that of the multifocal and diffuse types. This may be because the clinical symptoms of solitary nodule type HEH are not obvious; hence, it is difficult to detect. Certain studies have indicated that sub-marginal nodular lesions may be an early form of HEH, as they later gradually transform into the diffuse type [20]. These three patterns may represent different stages of disease progression. In addition, most of the lesions displayed well-defined, round-like, low-density nodules, with a trend of distribution in perihepatic regions. Regarding diffuse type HEH, there were often diffuse lesions of different sizes and ill-defined boundaries in the liver, and there was almost no normal liver parenchyma. These lesions often combine to form larger confluent masses. In our study, one patient with diffuse type HEH was misdiagnosed with hepatic fibrosis with a sub-marginal ‘strip-like’ sign accompanied by liver morphology abnormalities and proportion imbalance. Baron et al. reported these CT features [21, 22]. These signs are similar to liver cirrhosis, but there was no underlying liver disease in that case. Some diffuse type lesions in LM also showed strip-like enhancement, but there were no morphological changes in the liver and no obvious distribution characteristics. In 13 patients with HEH (13/20, 65%), retraction or flattening of the underlying liver capsule was observed. This percentage was higher than that reported by Zhao et al. [23], who found capsular retraction in 59.5% of HEH lesions in Chinese patients. Hepatic capsule retraction may be due to fibrous contraction in the central region of the lesion. In fact, approximately 2 to 2.8% of liver tumours may show this sign, including LM, intrahepatic cholangiocarcinoma, and fibrolamellar hepatocellular carcinoma, among others [24]. Some LMs after systemic chemotherapy or the primary tumours themselves, such as colon cancer, breast cancer, and carcinoid tumours, contain fibroses, displaying capsule retraction. In this study, the number of patients with hepatic capsule retraction in the LM group (6/60, 10%) was significantly lower than that in the HEH group. In addition, peripheral cholangiocarcinoma may cause capsular retraction due to an abundant fibrous stroma and chronic peripheral biliary obstruction. At present, these findings still need to be combined with other signs, including peripheral biliary dilatation, delayed diffuse heterogeneous enhancement, and a low probability of the multifocal type. Two HEH patients (2/20, 10%) showed small spots and nodule-like calcifications. This was lower than in previous reports, in which 15%-25% of patients had intralesional calcifications [25, 26]. However, some patients with LM from colon cancer also had calcification. Therefore, intralesional calcification is another important but nonspecific feature that contributes to the diagnosis of HEH.

Based on dynamic enhancement scanning, 10 patients (10/20, 50%) had minimal to slightly homogeneous enhancement and 9 (9/20, 45%) mainly thin rink-like enhancement in the arterial phase. There was no significant difference compared with LM. Interestingly, lesions with ring-like and mild enhancement can exist in the same patient. In the portal phase, most lesions exhibited slight to moderate, gradually homogeneous enhancement, with some large lesions (diameter larger than 3 cm) having heterogeneous enhancement. This is similar to the reports of Zhou et al. [27] and Klinger et al. [28]; indeed, this kind of enhancement characteristic in HEH is often misdiagnosed as LM. However, in LM, the rings are mostly thicker walled, more poorly defined, and obviously heterogeneous. We also used quantitative indicators (ER, TNR) to analyse enhancement characteristics, possibly reducing the influence of machine differences and individual variation. The results confirmed that HEH is a tumour with a poor blood supply, with an enhancement degree in the arterial phase lower than that in the adjacent liver parenchyma. The AER and ATNR of the HEH group were lower than those of the LM group (*p* < 0.05). Although the AUC of the AER was similar to that of the ATNR, the latter had higher sensitivity and specificity. Therefore, the ATNR was more valuable for differential diagnosis of LM from HEH. These differences in the above enhancement characteristics and ratios are usually based on individual histopathologic characteristics. Peripheral proliferation of HEH remains active, and a large number of arterial-venous shunts are formed. Tumour cells and stroma are present in variable proportions, and the central stromal portion of the lesion can vary from myxoid to densely fibrotic, which may account for the slight and thin rim-like enhancement in the arterial phase and hypo-enhancement in the portal phase. In LM, the blood supply of the tumour cells around the lesion is abundant, and the centre of the lesion is usually accompanied by haemorrhage and necrosis; thus, the range of ring-like enhancement is often larger and the degree of enhancement more obvious than that of HEH.

In addition, the typical imaging findings of some lesions in HEH, such as the “target” sign and “lollipop” sign, were seen in the portal phase. Mamone et al. [12] reported that the ‘‘target’’ sign is generated by central hypocellular, loose, fibrous myxoid stroma and necrotic tissue and is surrounded by viable, hypercellular, peripheral proliferating tumour cells. The outer narrow hypodense rim corresponds to the peripheral avascular zone caused by vascular infiltration or occlusion of hepatic sinusoids and small vessels. As the tumour grows, the central stroma gradually degenerates and becomes sclerotic as the blood supply decreases. In our study, a few LM lesions showed a “target” sign. We speculate that the centre of the tumour would bleed and necrose as the legion enlarges and show heterogeneous enhancement similar to the “target” sign when the inner edge is smooth. The solitary “target” sign-enhanced lesion needs to be distinguished from an abscess; the central density of the abscess is lower because of liquefaction and necrosis, though the edge of the lesion shows more obvious enhancement due to the proliferation of inflammatory cells, and abnormal perfusion often appears around the arterial phase. In general, high fever, chills and increased leucocyte counts may be helpful in diagnosing abscesses. Another typical feature of HEH, the “lollipop” sign was first reported by Alomari [29]. This sign presents as a well-defined peripherally enhancing (or non-enhancing) tumour mass with an avascular core on enhanced images (the “candy” in the “lollipop”) and the adjacent occluded vein (the stick) because HEH has a tendency to spread within the portal and hepatic vein branches. The vein should terminate smoothly at the edge or just within the rim of the lesion; vessels that traverse the entire lesion or are displaced, and collateral veins cannot be included in the sign. LM often directly invades adjacent blood vessels, resulting in wall stiffness, lumen stenosis or embolus formation. These two signs are conducive to distinguishing HEH from LM. Our study further confirmed the basic morphological features and typical signs of HEH described in the literature, but no other new signs were found.

Furthermore, HEH is often associated with extrahepatic organ invasion. In the study by Mehrabi et al., 36.6% of HEH patients had extrahepatic involvement, with organs such as the lung (8.5%), local lymph nodes (7.7%), peritoneum (6.1%), bone (4.9%), spleen (3.2%) and diaphragm (1.6%) as the most commonly involved sites [30]. Multipart CT scanning can be performed in one session to provide more information for an accurate diagnosis and comprehensive evaluation. In our study, 5 patients (25%) with HEH showed extrahepatic involvement, fewer than patients with LM. Regardless, it is difficult to differentiate the imaging manifestations of extrahepatic organ invasion.

The clinical prognosis of HEH is variable and unpredictable, ranging from an aggressive and fulminant course to possible long-term survival without definitive therapy [31]. The usual treatment for HEH includes liver resection, transcatheter arterial chemoembolisation, chemotherapy, radiation therapy and liver transplantation. Systemic treatment options include anti-angiogenic drugs such as pazopanib that inhibit HEH growth [32], immunotherapy with interferon α-2B, kinase inhibitors, and chemotherapy [33]. The prognosis of HEH is closely related to the size and number of lesions, the biological behaviour of the tumour (cellularity and cell necrosis), the degree of multiple organ damage, and the choice of treatment [10, 34]. According to our results, "target" sign has a moderate correlation with treatment results. Among 9 patients with the "target" sign of HEH, 8 cases were multiple and diffuse types; 6 cases progressed after treatment, which further indicates that tumour cells in the "target" sign lesion proliferate actively, with a poor prognosis. However, the overall survival rate of HEH patients was significantly higher than that of LM patients. Surgical resection is the best choice for the solitary nodular type with lesions confined to one liver lobe. Additionally, TACE is recommended if lesions are unresectable. Comprehensive treatment is more appropriate for HEH with multiple and diffuse types, though local resection easily leads to recurrence. In our study, 3 patients (3/4, 75%) showed a new lesion after resection, which is higher than in previous reports. This may be related to the pathological features of the lesion itself and the duration of follow-up. During follow-up, among the 3 patients who received interventional therapy, 2 had reduced lesions, and 1 showed no obvious change. Our findings also indicate that HEH is a low- to moderate malignant tumour and that proliferation of new tumour cells is slow after focal ischaemic necrosis. Regardless of extrahepatic involvement, liver transplantation is the most effective treatment for unresectable HEH [35]. In this study, no patients underwent liver transplantation, which may be related to their financial situation, understanding of the disease and other factors.

Our study has several limitations. First, because the study was retrospective, there was inevitable selection bias, especially in the primary diseases of LM. Nonetheless, liver metastases from different organs have some similar imaging features. We chose more primary tumour types according to the incidence of LM instead of certain tumour with equal numbers. Second, we set the ROI as the whole tumour rather than its periphery because most HEH lesions showed homogeneous minimal and slight enhancement, and it was difficult to define the active part around the tumour for some lesions with heterogeneous enhancement. However, whether the comparison of tumour periphery is meaningful for distinguishing HEH from LM needs further study. Third, most patients had multiple lesions with an average diameter of 3.85 cm, but we only selected 3 of them. To minimise selection bias, we limited the lesion diameter to 2–5 cm. Fourth, we used different types of CT scanners and protocols over the 8 years of the study. Nevertheless, we believe that morphological features may be unaffected by these nonconformities, and the application of ER and TNR may have greatly reduced their influence. Fifth, patients with different follow-up times had different prognoses. Larger studies are required to identify new signs associated with HEH and to confirm the present findings.

## Conclusions

In conclusion, dynamic CT imaging revealed special features of HEH, including subcapsular location and nodule coalescence, capsular retraction, intralesional calcifications, and slight and peripheral ring-like enhancement. When the typical “target” sign and “lollipop” sign are present, HEH should be highly suspected. CT imaging and a lower AER and TNR can, when combined with clinical data, help to improve differential diagnosis of HEH. Additionally, scanning multiple sites is conducive to accurate clinical grading and prognosis evaluation.

## Data Availability

The datasets used and/or analysed during the current study are available from the corresponding author on reasonable request.

## Abbreviations

AER: Arterial enhancement ratio
ATNR: Arterial tumour to normal parenchyma ratio
AUC: Areas under the curve
CIs: Confidence intervals
ER: Enhancement ratio
HEH: Hepatic epithelioid haemangioendothelioma
HU: Hounsfield units
LM: Liver metastasis
OR: Odds ratio
PER: Portal venous enhancement ratio
PTNR: Portal tumour to normal parenchyma ratio
RFA: Radio frequency ablation
ROC: Receiver operating characteristic
ROI: Regions of interest
TACE: Transcatheter arterial chemoembolisation
TNR: Ratio of tumour to normal parenchyma
